# Application of multimodal data fusion and intelligent classification in medical coding with the MCoder-T model

**DOI:** 10.1371/journal.pone.0338807

**Published:** 2026-01-07

**Authors:** Yisheng Li, Jie Zhao, Xinmei Li, Shanxiong Liang, Yihui Tang

**Affiliations:** Department of Medical Records Information, Guangxi Medical University Cancer Hospital, Nanning, Guangxi, China; Beijing Institute of Technology, CHINA

## Abstract

Assigning case codes is a complex problem in medical data processing, which includes multimodal data fusion and intellectual classification. Traditional case coding methods often have difficulties in managing different data sources and the complexity of the content of cases. This limits their effectiveness in actual application. To solve this problem, we propose the MCoder-T model, an intelligent case coding model, causal-to-mask attention mechanisms, integrated multimodal integration, and multi-task learning optimization. MCoder-T effectively improves case coding automation and classification accuracy by integrating text, medical images, and structured data. Experimental results show that the MCoder-T model outperforms traditional methods and other progressive models by several evaluation indicators, with an overall productivity improvement of 7% to 18%. The MCoder-T model enhances the automation of drop coding tasks and demonstrates reliable adaptability during multimodal data fusion and demonstrates broad application potential.

## Introduction

With the rapid development of information technology, data processing in the healthcare industry has entered the era of big data [1,2]. In this context, case coding plays a crucial role as a central element of medical information management. Coding cases is not only important for normalizing the diagnosis and treatment of diseases, but is also closely linked to the financial rules of hospitals and patient management [3]. However, traditional methods of coding cases are based on manual input, which is inefficient and prone to errors. As the types of medical data continue to grow, the burden of manual coding becomes increasingly heavy. Therefore, the automated technology of coding cases based on deep learning [4,5] has become a hot research topic, particularly in the field of multimodal data fusion and intelligent classification [6–9].

Significant progress has been made in recent years in Automation of coding case studies [10]. Traditional rules-based coding methods, while simple and intuitive, do not lack the flexibility and ability to Adaptation to complex data. This makes it inadequate for modern health environments [11,12]. Some studies have attempted to encode random texts using NLP methods such as vector word patterns, HMM, and CRF to extract disease codes from textual information [13]. However, most of these methods are based solely on textual data and do not take account of Importance of structured images and data [14]. With the widespread use of deep learning technologies such as CNN and RNN, a growing number of studies have begun integrating deep learning with case coding [15]. Some researchers attempt to incorporate image information into case coding by using CNN to extract features from medical images. But the problem of insufficient fusion of image and text information remains [16,17].

Many innovative solutions have been proposed for the problem of multimodal data connectivity [18,19]. Multimodal fusion methods based on GCN are widely used for the joint analysis of medical images and text data [20,21]. Other studies used DAE to reduce data size and extract characteristics, in combination with RL, to optimize classification strategies and ensure the common coding of text and structured data [22,23]. To process complex data, time series LSTM and GRU were used to predict diseases. However, these methods often ignore the information interaction between different methods [24]. Other methods, such as multimodal transformer-based processing frameworks [25], while effectively handling long-term dependencies, continue to have difficulties in properly managing the relationships between the different regimens in practical medical applications [26,27].

The combination of causal inference and multimodal fusion has become a new area of research in the field of medical data processing [28,29]. Many studies have introduced causation models to analyze causal links between different types of data, allowing more accurate assumptions of the disease [30]. For example, NCCs have been used to predict causal links between different medical events. However, they continue to encounter restrictions in the processing of multimodal data [31]. GNN-based models have also been proposed to establish links between images, text and other structured data. However, these methods have limited possibilities for processing incomplete or heavy data [32,33]. In addition, GAN is widely used to augment data and generate synthetic data to improve model reliability especially with small samples. However, their application in multimodal data fusion is still in progress study [34,35].

The aim of the article is to offer an innovative model for case coding, MCoder-T, which optimizes case coding automation through multimodal data fusion and intelligent classification using deep learning methods. Our work goes beyond traditional multimodal approaches to learning, such as the simple connection of properties, the fusion of tensors and standard intermodal attention, and introduces a more structured fusion strategy, which ensures accurate alignment of the properties while minimizing intermodal disturbances. The work presented in this document focuses on three main aspects: First, the MCoder-T model is introduced, effectively solving the problems of merging multimodal data and intelligent classification with the mechanism of masked cause and effect attention, solves the mechanism of the improved fusion PCMA and the optimization strategy MTL + LoRA. Secondly, the effectiveness of the MCoder-T model when testing the coding tasks for higher coding accuracy and a lower error rate, among other things. Finally, the paper explores the potential to extend the model to more areas of health data analysis in future research. Three key contributions to this document:

1. The MCoder-T model is proposed, based on multimodal deep learning and significantly improving the level of automation of case coding.
2. Integrating causal inferences and transmodal attention mechanisms improves the model’s ability to address complex dependencies between different data sources.
3. Experimental validation demonstrates the excellent performance of the MCoder-T on real health data sets and proves its feasibility in practical applications.

## Theory method

### Datasets

In the experiments presented in this document, we selected two data sets on medical records, which are accessible to the COVID-19 Radiography Database and MedMNIST. These datasets cover a wide range of applications, including lung imaging data and medical image classification tasks, and effectively support the fusion of multimodal data and medical image analysis in coding cases and tasks of intelligent classification. Table 1 summarizes the main characteristics of these two datasets.

**Table 1:** Key features and application scenarios of the datasets.

Dataset	Type	Number of Samples	Number of Classes	Format
COVID-19 Radiography Database	Chest X-ray Images	14,000 Images	Normal, Pneumonia, COVID-19	JPEG/PNG Images
MedMNIST	Medical Images	200,000 Images	Various Disease Categories	PNG Images

The COVID-19 Radiography Database contains a large number of chest X-ray images, primarily used for the diagnosis of COVID-19 [36]. The dataset includes three categories of labels: normal, pneumonia, and COVID-19. The diversity of image data and the definition of labels make it the best option for teaching and evaluating a deep learning model. The MedMNIST dataset focuses on the classification of medical images and contains multiple data pieces that solve various problems in the classification of diseases such as tuberculosis, covers breast cancer and netting diseases. Its wide image samples and various label categories provide broad data resources for case coding and intelligent classification models [37].

During data preprocessing, COVID-19 chest X-ray data bank line image was standardized, to ensure image size consistency for entry into a deep learning model for training. All images have been resized and normalized to a fixed resolution to eliminate differences in brightness and contrast between the images. Additionally, data extension techniques, including random rotation, translation, and scaling, were used to improve model robustness and simulate different shooting angles and patient position changes to increase model generalization capability. The MedMNIST dataset pre-processing process included similar steps. Standardized processing has been performed to resize each image, converting a grayscale, ensuring consistency of the input image data format, and eliminating changes caused by differences between devices. In order to increase the diversity of data sheets and improve training efficiency, data extension technologies such as random cuts or color changes are also used.

### Experimental details

In the experiment implemented in this document, a common deep learning framework for the education and evaluation of the model, especially PyTorch . Its flexibility and powerful computing capabilities effectively support multimodal modeling tasks. Experiment was implemented at a NVIDIA A100 high performance workstation equipped with a graphic processor. The processor is equipped to provide a large computer resource 32 GB RAM and Intel Xeon to manage large data sets and create models. All image data received a unified preprocessing, including variations, normalization, and data expansion. To improve drive stability, apply arbitrary cutting, rotation, and scaling to each image. This actually enhanced the diversity of data sets and improved the model’s capabilities. During training, the cross totropy loss function was used as an optimization target.

In order to ensure the reproducibility of the experiment and the stability of the results, all experiments were evaluated over several independent training periods. Each training session lasts about 10 hours, and the determined training duration depends on the size of the data sheet and the complexity of the model. During the training process, models were monitored, the results of the respective times were evaluated, check sets were used, and overruns were avoided by applying an early stop based on the accuracy of the check. After training, the model was evaluated in a test kit for final evaluation. All experiments were conducted in the same physical environment and setting to ensure the unfairness and consistency of the results.

### Evaluation metrics

In this section, in phar coding MCoder-T Five evaluation indicators were used to fully evaluate the performance of the model. These indicators evaluate models from several aspects such as classification accuracy, discrimination ability and recall rate. Error prediction and label pairing accuracy effectively reflect the overall performance of the model [38,39].

Subset accuracy is a commonly used measure in multi-label classification tasks. *N* represents the number of samples, *y*_*i*_ is the true label of the i-th sample, ${\hat{y}}_{i}$ is the intended label of the model. returns 1 if the predicted label corresponds exactly to the true one, otherwise 0. Subset accuracy measures the total compatibility of a model with multiple labeling tasks. The higher the value, the more accurate the prediction of the model.

$$\text{Subset Accuracy}=\frac{1}{N}\sum_{i=1}^{N}𝕀(y_{i}={\hat{y}}_{i})$$ (1)

Macro mean AUC-ROC are used to measure the discriminative ability of a model in a multiclass task. Average macro AUC Prevents class inequality from evaluating model performance. By providing a full representation of model performance in all categories. As the AUC value approaches 1, the stronger the model can distinguish between positive and negative samples.

$$\text{Macro AUC-ROC}=\frac{1}{C}\sum_{c=1}^{C}{AUC}_{c}$$ (2)

Macro Average F1 Score is a harmonic average between accuracy and recall. The F1 score takes into account both model accuracy and recall. *TP*_*i*_ is the true positive number of the digital label i, *FP*_*i*_ is the false number, *FN*_*i*_ is the false negative number. Macro Average F1 The scores are in all categories F1 average score. This measure allows for a complete assessment of the accuracy and revocability of the model in different categories and avoids prejudice against certain categories.

$${Precision}_{i}=\frac{TP_{i}}{TP_{i}+FP_{i}},\;{Recall}_{i}=\frac{TP_{i}}{TP_{i}+FN_{i}}$$ (3)

$$F1_{i}=2\times \frac{{Precision}_{i}\times {Recall}_{i}}{{Precision}_{i}+{Recall}_{i}}$$ (4)

$$\text{Macro F1 Score}=\frac{1}{C}\sum_{i=1}^{C}F1_{i}$$ (5)

Hamming loss measures the percentage of false label predictions in multiple label classification tasks. *L* represents the number of labels for each sample. *y*_*ij*_ is the true value of the j-th label for the i-th sample. ${\hat{y}}_{ij}$ is the level predicted by the model. 𝕀 is an index function that returns 1 if the predicted label is different from the actual label, and returns 0 if it is. The smaller the Hamming loss, the more accurate the model label will be.

$$\text{Hamming Loss}=\frac{1}{N}\sum_{i=1}^{N}\frac{1}{L}\sum_{j=1}^{L}𝕀(y_{ij}\neq {\hat{y}}_{ij})$$ (6)

The Jackcard Index is used to measure the similarity of planned and actual labels. The higher the Jackcard index, the higher the match between the model’s intended label and the actual label. For the multi-label classification task, the Jaccard Index effectively assesses the coverage capacity of the model’s labels.

$$J_{macro}=\frac{1}{N}\sum_{i=1}^{N}\frac{TP_{i}}{TP_{i}+FP_{i}+FN_{i}}$$ (7)

On these five evaluation metrics, you can fully and carefully evaluate the model’s performance in the MCoder-T case coding task. The combination of these measurements allows for a more modern understanding of the model’s strengths and weaknesses and provides the basis for further optimization.

## Method

### Overview of our network

**Fig 1:**
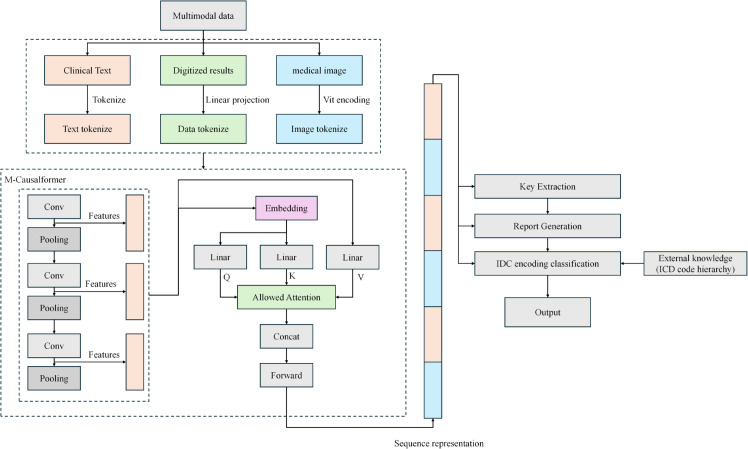
Architecture of the MCoder-T model for multimodal medical coding and intelligent classification.

The MCoder-T model is an intelligent fall coding and classification system based on multi-modal deplaning, and by integrating text, images and structured data improve the efficiency of automation and classification of fall coding. This model consists of three basic modules, as shown in Fig 1. This modules guarantees that the model can perform efficient and accurate classification tasks in the management of complex case data and at the same time resolve the main technical challenges of the multimodal data fusion solution.

The MCoder-T model integrates three main technical components to solve the problems of multimodal medical data processing. The Causal Mask Attention module uses a specialized attention mechanism that preserves time sequences in clinical data [40]. This component processes inputs by selectively focusing on the relevant characteristics of the different data types while maintaining the chronological integrity. In medical coding applications, this consistent treatment helps establish dependencies between different data sources and creates a solid foundation for further steps in coding analysis. The transmodal fusion module uses a structured approach to bringing together information from different sources of medical data. This component enables precise coordination of different data types through controlled interaction mechanisms [41]. The concept prevents common fusion problems such as data conflicts or feature degradations and allows each data module to effectively promote the overall representation while maintaining its distinctive properties. The Optimization Module incorporates dual strategies for model enhancement. The Multi-task learning enables simultaneously learn related clinical objectives and promote reliable functional recognition in the different areas of the tasks. Efficient for the parameters of fine-tuning technology reduces the calculation requirements when adapting the model and at the same time maintains the performance standards [42,43]. This combined approach ensures efficient operation for primary medical coding tasks while retaining flexibility for secondary clinical applications.

The MCoder-T model is based on modules based on these three technologies. The model shows excellent performance in case coding applications. The design of the model allows the processing of complex and multimodal data, improves both the accuracy and efficiency of coding and contributes greatly to the automation and intelligence of medical data processing.

### Causal masked attention module for multimodal data processing

**Fig 2:**
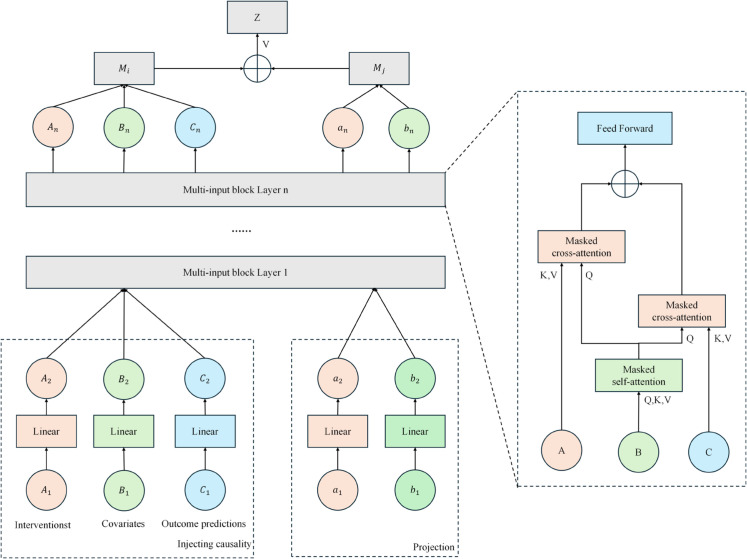
Architecture of the causal masked attention mechanism.

The Causal Mask Attention module is responsible for processing the input data of the main components of the MCoder-T model. It takes into account time dependencies and complex interactions between modalities in multimodal data using a M-Causalformer. Unlike the standard self-attention mechanism that allows all tokens to exercise global caution, our causal variant introduces a targeted limitation. The main feature of this module is to extract and protect efficient functions from multimodal data. These characteristics are correctly oriented and oriented by the mechanism of attention of the causal mask. This provides accurate information for subsequent merger and classification missions. Fig 2 shows the structure of this module.

The conventional self-attention mechanism calculates the relationship between each characteristic and all other characteristics in a bilateral way. which is not optimal for modeling sequential data where the future does not have to have an impact on the past. To address this, the causal mask attention mechanism limits the flow of information by introducing a mask matrix, ensuring that each token can interact only with previous tokens and itself during the calculation, thereby preserving the causal dependence in the data. *Q*, *K*, and *V* represent respectively a query, key and value matrix. $\frac{QK^{T}}{\sqrt{d_{k}}}$ calculates the similarity between features, softmax The function converts the similarity to weight. These weights are used to calculate the sum of the weights to get the output.

$$Attention(Q,K,V)=softmax\left(\frac{QK^{T}}{\sqrt{d_{k}}}\right)V$$ (8)

The key technical differentiator is the masked matrix M. This matrix plays a decisive role in the calculation of points of attention. It is defined as an upper triangular matrix with values of negative infinity −∞ in the positions that represent future tokens, effectively zeroing them out after the softmax operation. This ensures that each token can only attend to previous tokens and prevents any future information leakage. This stands in direct contrast to the standard self-attention, which applies no such restriction and uses a fully visible mask. This directed mechanism constrains the information flow and allows the model to learn causal dependencies during inference.

$$M_{ij}=\left\{ \begin{array}{cc} 0 & if\;i\geq j\\ -\infty & if\;i<j \end{array}\right.$$ (9)

After you enter the mask matrix, change the attention formula. The mask matrix *M* is added to the result of the similarity calculation to ensure and avoid the limitation of the cause mask, that future information enters the current information. Thus, the model guarantees the accuracy of cause and effect when processing multimodal data. Formally, the causal masked attention is computed by adding the mask matrix *M* to the scaled dot-product scores. This formulation ensures that the influence of future tokens is masked out, guaranteeing the causality of the model when processing multimodal data sequences.

$$Z=Attention(Q,K,V)=softmax\left(\frac{QK^{T}}{\sqrt{d_{k}}}+M\right)V$$ (10)

The output of the attention module into the cause mask is then processed step by step by a processing block, the step by step gains a deeper characteristic embodiment. These properties form a vector full of properties through intermodal interaction and fusion to provides detailed information for the following classification and coding tasks.

### Fine-grained cross-modal feature alignment and fusion with PCMA

**Fig 3:**
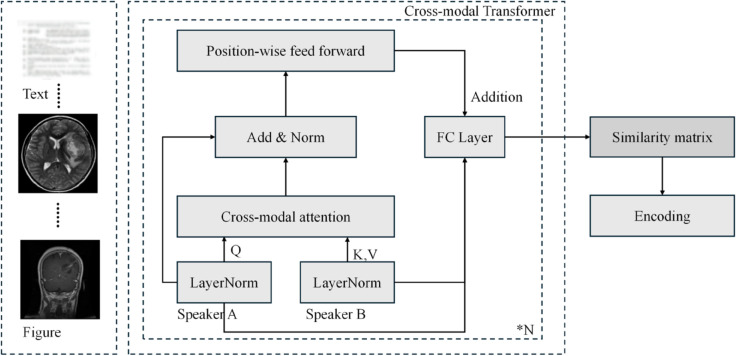
Architecture of the fine-grained cross-modal fusion module using PCMA.

The enhanced transmodal fusion module is a key component of the MCoder-T model. It combines PCMA technology and focuses on optimizing the orientation and interaction of the various modal data functions. This approach differs from conventional multimodal merger strategies. Unlike earlier fusion methods, such as simple concatenation, which often combats functional errors, or late fusion methods, which may not make sufficient use of intermodal correlations, our PCMA mechanism operates through structured intermediate fusion. In contrast to standard modal cross-attention that allows for unlimited flow of information, the PCMA introduces a pattern of controlled interaction through its "allowable attention mechanism" that effectively mitigates excess information and violations. This module solves the problem of multimodal data fusion, ensuring that different modalities contribute to complete functional information and do not disturb each other during the fusion process, improves the effect of multimodal data fusion and improves the accuracy of medical record coding tasks. Fig 3 shows the structural diagram of the module.

In this module, the fusion of transmodal features is performed through a PCMA transformer. First, the input feature is converted to a unified representation by linear transformation. The core innovation lies in the constrained cross-modal attention mechanism, which allows models to effectively capture relationships between various modalities only within carefully defined allowed pairs. *A* and *B* represent the index set of the pairing modality, and *P* represents the entire valid pairing relationship. This constraint ensures that any modality interaction occurs only in the allowed pairing, thereby preventing interference from irrelevant features.

AllowedAttention(A,B)={(i,j)∣i∈A,j∈B,(i,j)∈P} (11)

Another integration of the PCMA modular cross functions is carried out. *X*_*t*_ and *X*_*s*_ represent functions of different modes, while $head_{1},\ldots ,head_{h}$ represent the emission of several attention heads. *Concat* represents a bonding operation, *W*_*O*_ is the weight matrix. Multihead PCMA Through the parallel processing of several attention heads, modular transverse fusion is enhanced, learning the relationship between modalities from different points of view and enhancing the fusion effect.

$$\text{MultiHeadPCMA}(X_{t},X_{s})=LayerNorm\left(X_{t}+Concat(head_{1},\ldots ,head_{h})W_{O}\right)$$ (12)

After that PCMA operation is processed by position coding and the feed-forward network. *x* represents the input characteristic. *W*_1_ and *W*_2_ are the weight matrices, and *b*_1_ and *b*_2_ are the bias terms. ReLU Is an activation function. This process processes the features of each location to improve the ability to display information. Features facilitates learning and effective extraction of information.

$$\text{FeedForward}(X)=W_{2}\cdot ReLU(W_{1}\cdot X+b_{1})+b_{2}$$ (13)

This module enables the successful integration of functions of different modes thanks to modular functional interactions, position coding and layer normalization and provides a precise basis for optimization and classification of subsequent tasks.

### Integration of multi-task learning and LoRA for efficient model optimization

**Fig 4:**
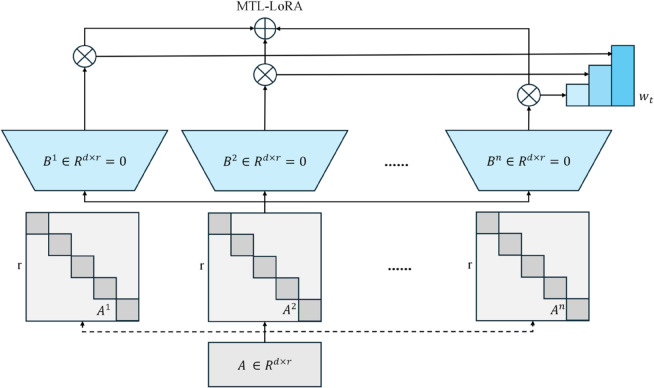
Architecture of the multi-task learning and LoRA integration module.

The multi-task optimization module combines MTL and LoRA technologies to improve model generalizability and optimization efficiency. In our multi-task learning framework, we design two specific auxiliary tasks alongside the primary medical coding task to enhance feature learning: a contrastive learning task that maximizes mutual information between different modalities of the same case while minimizing similarity across different cases, which improves cross-modal alignment; and a disease severity prediction task that classifies cases into mild, moderate, and severe categories based on clinical indicators, providing complementary clinical context to the main coding task. The main function of this module is to enable models to learn a more complete representation of features through multitasking learning. On the other hand, LoRA technology allows you to effectively correct large deep learning models with a low adjustment, by effectively reducing computing costs while maintaining high productivity. Fig 4 shows the structure of this module.

Multitask learning uses common model parameters, so that the model can optimize multiple tasks simultaneously. These auxiliary tasks are carefully selected to be clinically relevant, but differ from the main purpose of coding, so they provide additional learning signals and there is no contradictory optimization. In the process, LoRA optimizes the model by introducing a low-rank matrix and reduces computation costs. *B* and *A* respectively represent a low storage matrix and an upper projection matrix, ΔW indicates the update of the model parameters. The model parameters are adjusted using an updated low-rank matrix format and reduce computation complexity while maintaining performance. *W*_0_ represents the previously trained weight, *x* which represents the input data, ΔW=B·A·x is an updated LoRA parameter.

$$h=W_{0}\cdot x+\Delta W=W_{0}\cdot x+B\cdot A\cdot x$$ (14)

In MTL, we optimize the performance of multiple tasks through a joint loss function. The total loss function integrates the primary medical coding task with the auxiliary contrastive learning and severity prediction tasks, where each task contributes to the overall learning objective according to its designated weight. The number of the weight of the mission is $\lambda_{k}$. This multitasking optimization allows the model to learn more extensive features from different tasks and improve the generalization ability for each task.

$${ℒ}_{total}(\theta_{s},\theta_{1},\ldots ,\theta_{K})=\sum_{k=1}^{K}\lambda_{k}{ℒ}_{k}(\theta_{s},\theta_{k})$$ (15)

$$h=W_{0}\cdot x+(BA)x$$ (16)

In multi tasking learning, $\theta_{s}^{LoRA}$ represents a shared parameter that is refined. Thus MCoder-T The model can perform task optimization and precise low level adaptation at the same time. Efficiency and scalability in multi tasking environments were guaranteed.

$${ℒ}_{total}=\sum_{k=1}^{K}\lambda_{k}{ℒ}_{k}(\theta_{s}^{LoRA},\theta_{k})$$ (17)

Multitasking Learnen und LoRA through the combination of technologies, The MCoder-T model significantly improves performance for multiple tasks and reduces computation costs for model adjustment. This module ensures that the model works efficiently for case coding tasks and can be extended to other related tasks, has improved both overall performance and efficiency.

## Experiment

### Comparative experiments and analysis

In this section, the experiments of MCoder-T and everal comparison models on Explore several comparative models of the COVID-19 and MedMNIST dataset and provide a detailed analysis of the results. Using five key evaluation metrics, we compare the performance of the MCoder-T model with other comparison models. Table 2 presents the experimental results.

**Table 2:** Performance comparison under standard and robustness settings on COVID-19 and MedMNIST datasets.

Model	Dataset	Setting	Subset Accuracy(%)	Macro F1(%)	Macro AUC-ROC	Jaccard Index(%)	Hamming Loss(%)
MCoder-T	COVID-19	Standard	55.2	64.8	91.2	60.1	3.4
		+20% Noise	53.1	63.2	90.1	58.3	3.7
		-30% Data	52.8	62.9	89.8	58.0	3.8
	MedMNIST	Standard	56.0	65.5	92.0	60.8	3.0
		+20% Noise	54.3	64.1	91.2	59.2	3.3
		-30% Data	54.0	63.8	90.9	58.9	3.4
MedConGTM [44]	COVID-19	Standard	49.0	61.0	88.0	56.0	4.5
		+20% Noise	45.2	58.3	85.1	53.1	5.2
	MedMNIST	Standard	49.5	61.5	88.5	57.0	4.8
		+20% Noise	46.1	59.0	86.3	54.5	5.4
CRAKUT [45]	COVID-19	Standard	51.1	62.5	87.5	58.0	4.2
		+20% Noise	47.8	60.1	84.9	55.8	4.8
	MedMNIST	Standard	51.5	63.0	88.0	59.0	4.4
		+20% Noise	48.3	60.8	85.7	56.9	5.0
GCFormer [46]	COVID-19	Standard	-	63.5	-	59.0	4.1
		+20% Noise	-	60.8	-	56.5	4.6
	MedMNIST	Standard	-	64.0	-	59.5	4.3
		+20% Noise	-	61.5	-	57.2	4.8
DRCNN-ATT [47]	COVID-19	Standard	-	64.0	-	59.5	3.8
		+20% Noise	-	61.2	-	57.0	4.3
	MedMNIST	Standard	-	64.5	-	60.0	3.9
		+20% Noise	-	61.8	-	57.8	4.4
LLaMA-3 [48]	COVID-19	Standard	50.0	61.5	87.0	57.0	4.6
		+20% Noise	46.5	58.9	84.2	54.2	5.2
	MedMNIST	Standard	51.0	62.5	87.5	58.0	5.0
		+20% Noise	47.8	60.1	85.0	55.5	5.6

**Fig 5:**
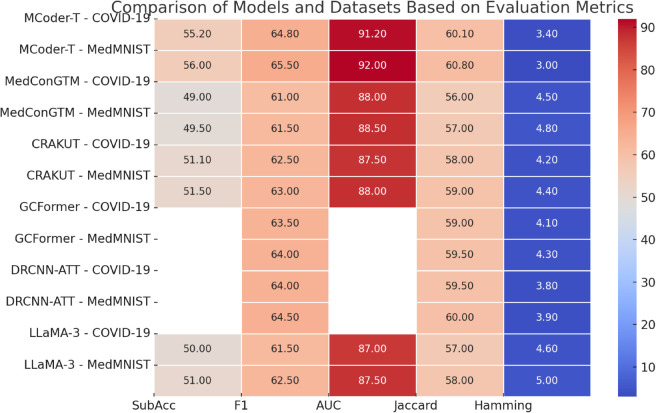
Performance comparison of MCoder-T and baseline models under standard settings.

As shown in Fig 5, the MCoder-T model significantly outperforms other comparison models on both the COVID-19 and MedMNIST datasets. Initially, MCoder-T achieved 55.2% of the total COVID-19 dataset in terms of subset accuracy and improved by approximately 12.7% (49.0%) compared to MedConGTM. Across the MedMNIST dataset, he showed an improvement of 9.8%, from 51.0% with LLaMA-3 to 56.0%. This result shows that MCoder-T can better recognize multi-labels, reduce missed predictions, and improve classification accuracy. Regarding the macro mean F1, MCoder-T reached 64.8% in the overall COVID-19 dataset, representing a 3.7% improvement over CRAKUT (62.5%). and represents a 3.2% improvement in the overall MedMNIST data set from 63.0% in CRAKUT to 65.5%. The F1 rating is a harmonious average accuracy and memory, and the MCoder-T is characterized by a balance of these two aspects while providing accuracy and memory in managing multi-label issues. For macro-average AUC-ROC, MCoder-T reached 91.2% across the COVID-19 dataset, an improvement of approximately 3.2% compared to MedConGTM (88.0%). Across the MedMNIST dataset, MCoder-T outperformed LLaMA-3 (87.5%) by 4.5%, demonstrating its ability to perform more precise differences in multi-class tasks. especially for tasks for coding complex cases in which it better recognizes different categories. Regarding the Jaccard index, MCoder-T scored 60.1% in the overall COVID-19 dataset, which is 7.3% higher than MedConGTM (56.0%). According to the entire MedMNIST dataset, the Jaccard MCoder T-index was 60.8%, which is 5.1% better than LLaMA-3 (58.0%). The Jaccard index reflects the ability to match the model’s labels, and an improvement in this index shows that the MCoder-T can match the labels more accurately. For Hamming loss, which measures the number of mispredicted markers, MCoder-T reached 0.034 across the COVID-19 dataset, which is 26.1% less than LLaMA-3 (0.046). After the entire MedMNIST dataset, it reduced losses by 40%. A lower Hamming loss means that MCoder-T has a lower error rate in label prediction, reducing the number of mispredicted labels and further improving the accuracy of case coding.

In addition to the standard performance evaluation, we further investigated the model’s robustness to address practical clinical requirements. As shown in Table 2, MCoder-T demonstrates superior resilience against noisy inputs and missing data compared to baseline models. Under the +20% noise condition, MCoder-T maintains stable performance across both datasets, with only marginal performance degradation (e.g., Macro F1 score decreases by 1.6% on COVID-19 and 1.4% on MedMNIST), whereas other models exhibit significantly larger performance drops. This robustness can be attributed to our architectural design: the mask’s causal attention mechanism effectively filters out insignificant noise samples, limiting information flows, while the PCMA fusion module by its controlled interaction scheme prevents the spread of errors on the modules. In addition, MCoder-T maintains competitive performance in case of -30% data shortage and verifies its ability to process incomplete clinical records. These results together confirm that MCoder-T not only achieves exceptional accuracy, but also possesses important resistance properties for real-world clinical application.

**Fig 6:**
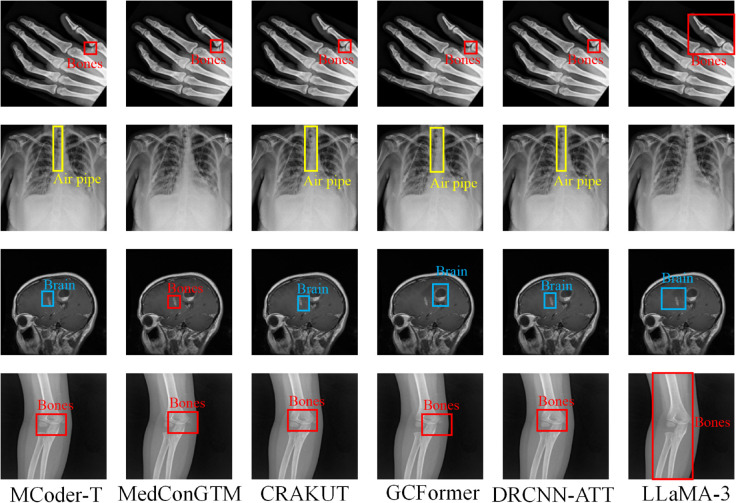
Comparison of experimental results for MCoder-T and baseline models.

As in Fig 6, MCoder-T consistently outperforms all comparative models, particularly in the area of merging multimodal data and optimizing multitasking learning. This suggests that MCoder-T can effectively manage data from different modes, can make full use of information from any fashion and avoid problems of information loss and inconsistency, thereby significantly improving the performance of the coding task.

### Ablation experiments and analysis

To further verify the contribution of each module to the MCoder-T model, we conducted ablation experiments by removing individual modules and studying their impact on overall performance [49]. Experimental data are presented in Table 3. By comparing indicator changes before and after the removal of different modules, we can clearly define the improvement and optimization of each module’s impact on performance observe.

**Table 3:** Ablation study results of MCoder-T model by removing individual modules.

Model	Dataset	Subset Accuracy (%)	Macro F1 (%)	Macro AUC-ROC	Jaccard Index (%)	Hamming Loss (%)
MCoder-T	COVID-19	55.2	64.8	91.2	60.1	3.4
	MedMNIST	56.0	65.5	92.0	60.8	3.0
W/o Causal Masked Attention	COVID-19	51.1	62.1	87.5	56.3	4.0
	MedMNIST	51.5	62.5	88.0	57.0	4.2
W/o PCMA Fusion	COVID-19	52.3	62.8	89.0	57.8	3.9
	MedMNIST	52.7	63.3	89.5	58.2	4.1
W/o Multi-task Learning Optimization	COVID-19	52.9	63.2	88.5	58.0	3.8
	MedMNIST	53.2	63.7	89.0	58.5	4.0

**Fig 7:**
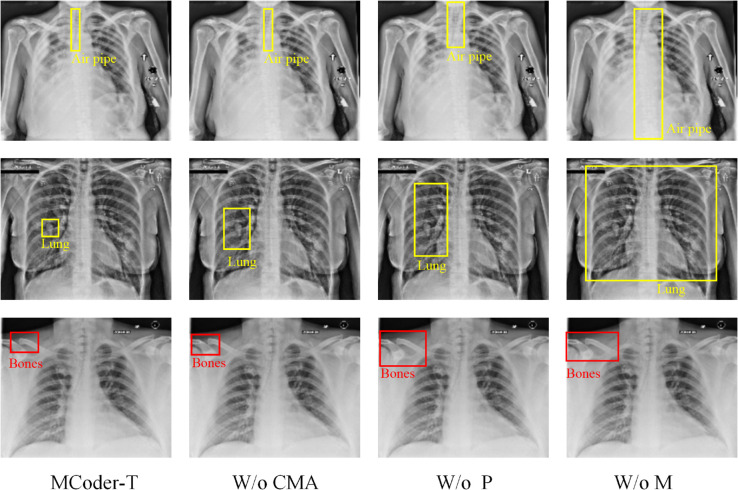
Effect of MCoder-T model after module ablation.

The experimental results show that after removing the attention module from the cause-effect mask, the performance of the model in all indicators, including the subset accuracy, macro-average F1 score, macro-average AUC-ROC, Jacques index, and Hamming loss. For example, after removing this module, the accuracy of the entire COVID-19 data set decreased from 55.2% to 51.1%, representing a decrease of about 7.4%. Also the average macro F1 score fell from 64.8% to 62.1%. This power balance shows, that the attention module of the causal mask plays an important role in detecting the causal dependence between the modalities and reinforcing the multimodal data fusion played. After removing the module, model performance also decreased across all metrics, especially in macro averages. AUC-ROC decreased from 91.2% to 89.0% and decreased by approximately 2.4%. This result indicates that the PCMA module plays a key role in fine grain fusion of features, effectively aligns and interacts with multimodal data, avoids information loss, and improves model performance by distinguishing different categories. After the removal of the multitask learning optimization module, model productivity decreased, particularly with the Jacques Index and Hamming loss. The Jacques index fell from 60.1% to 58.0%, down 3.5%. While multitask learning improves the model’s ability to generalize different tasks, its impact on the case coding task is relatively small. Also after deleting this module, the model retained some performance, but lost the advantages of multitask optimization, which shows that this module is important for processing multitask scenarios. Fig 7 shows the results of the tests carried out after the module has been removed.

However, although ablation experiments on individual modules can confirm the independent contribution of each module, they do not fully show synergy effects between the modules. To further test the coherence and collaboration between the modules, we carried out remote experiments with several modules, to evaluate the synergy effects of module optimization in the task of multimodal data fusion and case coding [50]. Table 4 shows the experimental results after ablation of several modules.

**Table 4:** Ablation study results of MCoder-T model by removing multiple modules.

Model	Dataset	Subset Accuracy (%)	Macro F1 (%)	Macro AUC-ROC	Jaccard Index (%)	Hamming Loss (%)
MCoder-T	COVID-19	55.2	64.8	91.2	60.1	3.4
	MedMNIST	56.0	65.5	92.0	60.8	3.0
W/o Causal Masked Attention + PCMA	COVID-19	50.5	61.3	87.8	55.8	4.1
	MedMNIST	51.0	61.5	88.2	56.2	4.3
W/o Causal Masked Attention + MTL Optimization	COVID-19	51.2	62.0	88.0	56.5	4.0
	MedMNIST	51.7	62.3	88.5	56.8	4.2
W/o PCMA + MTL Optimization	COVID-19	52.0	62.5	88.3	57.0	3.9
	MedMNIST	52.5	62.8	88.8	57.2	4.0

**Fig 8:**
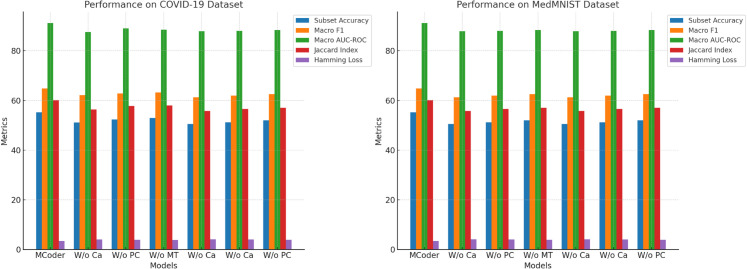
Ablation study results of multiple modules in MCoder-T model for investigating the interaction of module combinations.

As shown in Fig 8, after the elimination of the mask causal attention module and the PCMA module, the performance of the model decreased significantly on all evaluation indicators. in particular in the accuracy of the subset and the macro mean F1 score. According to the COVID-19 database, subset accuracy after deletion of these two modules decreased from 55.2% to 50.5%, or about 8.5%. According to MedMNIST, it decreased by about 8.9%, from 56.0% to 51.0%. This indicates that the The absence of these modules significantly reduces the fusion capacity of the information, which affects model productivity for multi-label classification tasks. If the mask causal attention module and the MTL optimization module are removed, model performance decreases, particularly with the macromean AUC-ROC and the Jacques index. According to the COVID-19 database, the macromean AUC-ROC decreased from 91.2% to 88.0%, or approximately 3.5%. In addition, the decrease in the MedMNIST dataset has is about 3.8%, from 92.0% to 88.2%. This decrease in performance shows the critical role that the MTL optimization module plays in the overall performance of the model, particularly in multitask scenarios in which it assists the model optimization for all tasks. In addition, the deletion of the PCMA module and the MTL optimization module has resulted in power fluctuations, in particular in the average macro index F1 and the Jacques index. According to the COVID-19 dataset, the mean macroindex F1 decreased from 64.8% to 62.5%, a decrease of approximately 3.6%. For MedMNIST, the decrease was 3.7% from 65.5% to 62.8%. These results show that the optimization module PCMA and the optimization module MTL have a significant impact on the efficient merging of the functions of the different modes and the optimization of multitask productivity. Without this module, the model struggles with complex tasks.

Through multi-modular ablation experiments, we have confirmed the importance of each module in the MCoder-T model and demonstrated the synergy effects between these modules. The collaboration of these modules is crucial for the effective management of numerous modeling tasks and the precise prediction of coding cases. Therefore, the removal of each module has a significant impact on the overall model performance and confirms the effectiveness of the MCoder-T model development and optimization strategy in Tasks of multimodal learning.

## Conclusion

In this article, we propose the MCoder-T model, a case coding and intelligent classification system based on multimodal deep learning. By combining the causal M-Causalformer, PCMA, and MTL+LoRA, MCoder-T integrates text, images and structured data efficiently and improves automation efficiency and classification accuracy of case coding tasks. Through a number of comparative and ablation experiments, we have demonstrated the important advantages of the model over existing models across several evaluations, particularly in multimodal data fusion, model accuracy, and computational efficiency. Experimental results show that MCoder-T not only ensures efficient operation when performing coding tasks, but also demonstrates a strong generalization capability. It can be extended to other related tasks.

Future research aims to further optimize the integration of multimodal functions and the possibilities of joint learning of tasks of the MCoder-T model. be. In particular, with regard to learning efficiency and real-time performance of the model in large-scale multitasking environments, Future work could introduce more methods of self-controlled learning and dispersion strategies, to further increase the speed of model conclusions and the efficiency of calculations. The MCoder-T model will be applied to a larger number of medical records and real clinical scenarios, to test its effectiveness in practical applications and to investigate how to model smarter healthcare solutions such as supportive diagnostics, can provide personalized treatment and health management [51,52].

## References

[1] Soenksen LR, Ma Y, Zeng C, Boussioux L, Villalobos Carballo K, Na L, et al. Integrated multimodal artificial intelligence framework for healthcare applications. NPJ Digit Med. 2022;5(1):149. doi: 10.1038/s41746-022-00689-4 36127417 PMC9489871

[2] Shynar Y, Seitenov A, Kenzhegarina A, Kenzhetayev A, Kemel A, Ualiyev N, et al. Comprehensive analysis of blockchain technology in the healthcare sector and its security implications. International Journal of E-Health and Medical Communications. 2025;16(1):1–45. doi: 10.4018/ijehmc.372423

[3] Ji S, Li X, Sun W, Dong H, Taalas A, Zhang Y, et al. A unified review of deep learning for automated medical coding. ACM Comput Surv. 2024;56(12):1–41. doi: 10.1145/3664615

[4] Li S, Hu J, Zhang B, Ning X, Wu L. Dynamic personalized federated learning for cross-spectral palmprint recognition. IEEE Trans Image Process. 2025;34:4885–95. doi: 10.1109/TIP.2025.3592508 40737152

[5] Hao M, Gu Y, Dong K, Tiwari P, Lv X, Ning X. A prompt regularization approach to enhance few-shot class-incremental learning with two-stage classifier. Neural Netw. 2025;188:107453. doi: 10.1016/j.neunet.2025.107453 40220563

[6] Lahat D, Adali T, Jutten C. Multimodal data fusion: an overview of methods, challenges, and prospects. Proc IEEE. 2015;103(9):1449–77. doi: 10.1109/jproc.2015.2460697

[7] Qiu Q, Pei C, Sun R, Liu B. Mitigating safety risks via integrated proactive backup and mission abort policies. Reliability Engineering & System Safety. 2026;265:111445. doi: 10.1016/j.ress.2025.111445

[8] Qiu Q, Liu B, Pei C, Sun R, Zhao X. Optimal condition-based backup and mission abort decisions for cloud computing systems. Quality and Reliability Engineering International. 2025.

[9] Qiu Q, Maillart LM, Prokopyev OA, Cui L. Optimal condition-based mission abort decisions. IEEE Trans Rel. 2023;72(1):408–25. doi: 10.1109/tr.2022.3172377

[10] Kodipalli A, Fernandes SL, Dasar SK, Ismail T. Computational framework of inverted fuzzy C-means and quantum convolutional neural network towards accurate detection of ovarian tumors. International Journal of E-Health and Medical Communications. 2023;14(1):1–16. doi: 10.4018/ijehmc.321149

[11] Almarshadi NF, Alharbi MMA, Alharbi MS, Alwaked WA, Alsayer HM, Alhussain SM. Clinical and medical coding: a new pathway for automation-an updated review. Journal of Medical and Life Science. 2024;6(4):633–51. doi: 10.21608/jmals.2024.413890

[12] Qiu Q, Kou M, Chen K, Deng Q, Kang F, Lin C. Optimal stopping problems for mission oriented systems considering time redundancy. Reliability Engineering & System Safety. 2021;205:107226. doi: 10.1016/j.ress.2020.107226

[13] Khurana D, Koli A, Khatter K, Singh S. Natural language processing: state of the art, current trends and challenges. Multimed Tools Appl. 2023;82(3):3713–44. doi: 10.1007/s11042-022-13428-4 35855771 PMC9281254

[14] Huang Z, Xiao Z, Ao C, Guan L, Yu L. Computational approaches for predicting drug-disease associations: a comprehensive review. Front Comput Sci. 2024;19(5). doi: 10.1007/s11704-024-40072-y

[15] Masud JHB, Kuo C-C, Yeh C-Y, Yang H-C, Lin M-C. Applying deep learning model to predict diagnosis code of medical records. Diagnostics (Basel). 2023;13(13):2297. doi: 10.3390/diagnostics13132297 37443689 PMC10340491

[16] Shetty S, S AV, Mahale A. Multimodal medical tensor fusion network-based DL framework for abnormality prediction from the radiology CXRs and clinical text reports. Multimed Tools Appl. 2023;:1–48. doi: 10.1007/s11042-023-14940-x 37362656 PMC10119019

[17] Cao J, Zou Q, Ning X, Wang Z, Wang G. FIPNet: self-supervised low-light image enhancement combining feature and illumination priors. Neurocomputing. 2025;623:129426. doi: 10.1016/j.neucom.2025.129426

[18] Xing X, Wang B, Ning X, Wang G, Tiwari P. Short-term OD flow prediction for urban rail transit control: a multi-graph spatiotemporal fusion approach. Information Fusion. 2025;118:102950. doi: 10.1016/j.inffus.2025.102950

[19] Miao J, Ning X, Hong S, Wang L, Liu B. Secure and efficient authentication protocol for supply chain systems in artificial-intelligence-based Internet of Things. IEEE Internet Things J. 2025;12(19):39532–42. doi: 10.1109/jiot.2025.3592401

[20] Wang L, Zhang J, Liu Y, Mi J, Zhang J. Multimodal medical image fusion based on gabor representation combination of multi-CNN and fuzzy neural network. IEEE Access. 2021;9:67634–47. doi: 10.1109/access.2021.3075953

[21] Fang L, Deng Q, Yang D. Integration of multi-domain das data analysis and machine learning for wellbore flow regimes identification in shale gas reservoirs. Energy. 2025;332:137123. doi: 10.1016/j.energy.2025.137123

[22] Kiraz AH, Djibrillah FO, Yüksel ME. Deep feature extraction, dimensionality reduction, and classification of medical images using combined deep learning architectures, autoencoder, and multiple machine learning models. Turkish Journal of Electrical Engineering and Computer Sciences. 2023;31(6):1113–28. doi: 10.55730/1300-0632.4037

[23] Gao W, Li X, Wang Y, Cai Y. Medical image segmentation algorithm for three-dimensional multimodal using deep reinforcement learning and big data analytics. Front Public Health. 2022;10:879639. doi: 10.3389/fpubh.2022.879639 35462800 PMC9024167

[24] Rajmohan R, Pavithra M, Kumar TA, Manjubala P. Exploration of deep RNN architectures: LSTM and GRU in medical diagnostics of cardiovascular and neuro diseases. Handbook of Deep Learning in Biomedical Engineering and Health Informatics. Apple Academic Press; 2021. p. 167–202.

[25] Li J, Wang Y, Ning X, He W, Cai W. FefDM-transformer: dual-channel multi-stage transformer-based encoding and fusion mode for infrared–visible images. Expert Systems with Applications. 2025;277:127229. doi: 10.1016/j.eswa.2025.127229

[26] Song S, Li X, Li S, Zhao S, Yu J, Ma J, et al. How to bridge the gap between modalities: survey on multimodal large language model. IEEE Trans Knowl Data Eng. 2025;37(9):5311–29. doi: 10.1109/tkde.2025.3527978

[27] Bai R, Bai B. The impact of labor productivity and social production scale on profit-induced demand: function and analysis from the perspective of Marx’s economic theory. Journal of Xi’an University of Finance and Economics. 2024;37(5):3–17. doi: 10.19331/j.cnki.jxufe.20240802.001

[28] Holzinger A, Malle B, Saranti A, Pfeifer B. Towards multi-modal causability with graph neural networks enabling information fusion for explainable AI. Information Fusion. 2021;71:28–37. doi: 10.1016/j.inffus.2021.01.008

[29] DeMatteo C, Jakubowski J, Stazyk K, Randall S, Perrotta S, Zhang R. The headaches of developing a concussion app for youth. International Journal of E-Health and Medical Communications. 2024;15(1):1–20. doi: 10.4018/ijehmc.352514

[30] Jiao L, Wang Y, Liu X, Li L, Liu F, Ma W, et al. Causal inference meets deep learning: a comprehensive survey. Research (Wash D C). 2024;7:0467. doi: 10.34133/research.0467 39257419 PMC11384545

[31] Taghados Z, Azimifar Z, Monsefi M, Jahromi MA. CausalCervixNet: convolutional neural networks with causal insight (CICNN) in cervical cancer cell classification-leveraging deep learning models for enhanced diagnostic accuracy. BMC Cancer. 2025;25(1):607. doi: 10.1186/s12885-025-13926-2 40181353 PMC11969838

[32] Waqas A, Tripathi A, Ramachandran RP, Stewart PA, Rasool G. Multimodal data integration for oncology in the era of deep neural networks: a review. Front Artif Intell. 2024;7:1408843. doi: 10.3389/frai.2024.1408843 39118787 PMC11308435

[33] Almayyan WI, AlGhannam BA. Detection of kidney diseases: importance of feature selection and classifiers. International Journal of E-Health and Medical Communications. 2024;15(1):1–21.

[34] Biswas A, Md Abdullah Al N, Imran A, Sejuty AT, Fairooz F, Puppala S, et al. Generative adversarial networks for data augmentation. Data Driven Approaches on Medical Imaging. Springer; 2023. p. 159–77. 10.1007/978-3-031-47772-0_8

[35] Deng Q, Jiang J, Yang D, Han H, Qi G. Dynamic analysis and optimization of perforated tubing strings in deep-water wells under diverse operating conditions. Ocean Engineering. 2025;322:120535. doi: 10.1016/j.oceaneng.2025.120535

[36] Bhosale YH, Patnaik KS. Application of deep learning techniques in diagnosis of covid-19 (coronavirus): a systematic review. Neural Process Lett. 2022:1–53. doi: 10.1007/s11063-022-11023-0 36158520 PMC9483290

[37] Rajoria R, Kanodia B, Saha D, Singh PK. A deep feature ensemble methodology for 2D biomedical image classification. In: International Conference on Computational Intelligence in Pattern Recognition. Springer; 2024. p. 85–99.

[38] Al-Zoghby AM, Ismail Ebada A, Saleh AS, Abdelhay M, Awad WA. A comprehensive review of multimodal deep learning for enhanced medical diagnostics. Computers, Materials & Continua. 2025;84(3).

[39] Shamshirband S, Fathi M, Dehzangi A, Chronopoulos AT, Alinejad-Rokny H. A review on deep learning approaches in healthcare systems: taxonomies, challenges, and open issues. J Biomed Inform. 2021;113:103627. doi: 10.1016/j.jbi.2020.103627 33259944

[40] Sanchez P, Voisey JP, Xia T, Watson HI, O’Neil AQ, Tsaftaris SA. Causal machine learning for healthcare and precision medicine. R Soc Open Sci. 2022;9(8):220638. doi: 10.1098/rsos.220638 35950198 PMC9346354

[41] Wang J, Zhu L, Yu X, Bhalerao A, He Y. Improving medical visual representation learning with pathological-level cross-modal alignment and correlation exploration. arXiv preprint 2025. https://arxiv.org/abs/250610573 10.1109/JBHI.2025.3624382 41129431

[42] Agiza A, Neseem M, Reda S. Mtlora: low-rank adaptation approach for efficient multi-task learning. In: Proceedings of the IEEE/CVF conference on computer vision and pattern recognition; 2024. p. 16196–205.

[43] Zhang L, Liu J, Wei Y, An D, Ning X. Self-supervised learning-based multi-source spectral fusion for fruit quality evaluation: a case study in mango fruit ripeness prediction. Information Fusion. 2025;117:102814. doi: 10.1016/j.inffus.2024.102814

[44] Çelikten T, Onan A. Medcongtm: Interpretable multi-label clinical code prediction with dual-view graph contrastive topic modeling. Knowledge-Based Systems. 2025;327:114103. doi: 10.1016/j.knosys.2025.114103

[45] Liang Y, Zhu X, Huang M, Zhang W, Guo H, Feng Q. CRAKUT:integrating contrastive regional attention and clinical prior knowledge in U-transformer for radiology report generation. Nan Fang Yi Ke Da Xue Xue Bao. 2025;45(6):1343–52. doi: 10.12122/j.issn.1673-4254.2025.06.24 40579148 PMC12204827

[46] Yu W, Zhuo L, Li J. GCFormer: global context-aware transformer for remote sensing image change detection. IEEE Trans Geosci Remote Sensing. 2024;62:1–12. doi: 10.1109/tgrs.2024.3381738

[47] Bhutto SR, Zeng M, Niu K, Khoso S, Umar M, Lalley G, et al. Automatic ICD-10-CM coding via lambda-scaled attention based deep learning model. Methods. 2024;222:19–27. doi: 10.1016/j.ymeth.2023.11.017 38141869

[48] Dubey A, Jauhri A, Pandey A, Kadian A, Al-Dahle A, Letman A, et al. The llama 3 herd of models. arXiv e-prints. 2024.

[49] Sheikholeslami S, Meister M, Wang T, Payberah AH, Vlassov V, Dowling J. Autoablation: Automated parallel ablation studies for deep learning. In: Proceedings of the 1st workshop on machine learning and systems. 2021. p. 55–61.

[50] Kumar S, Rani S, Sharma S, Min H. Multimodality fusion aspects of medical diagnosis: a comprehensive review. Bioengineering (Basel). 2024;11(12):1233. doi: 10.3390/bioengineering11121233 39768051 PMC11672922

[51] Huang J, Chen J, Lv L, Yu Z, Yao W, Cheng H, et al. Design and verification of a wearable microcapacitance test system for POC biosensing. IEEE Trans Instrum Meas. 2025;74:1–11. doi: 10.1109/tim.2025.3555726

[52] Qi H, Hu Z, Yang Z, Zhang J, Wu JJ, Cheng C, et al. Capacitive aptasensor coupled with microfluidic enrichment for real-time detection of trace SARS-CoV-2 nucleocapsid protein. Anal Chem. 2022;94(6):2812–9. doi: 10.1021/acs.analchem.1c04296 34982528

